# Supplementary material for: Bergamot Polyphenols Reduce Hepatic Lipogenesis While Boosting Autophagy and Short-Chain Fatty Acid Production in a Murine “Cafeteria” Model of MASLD
Source: Nutrients. 2025 Nov 25;17(23):3684. doi: 10.3390/nu17233684 (PMC12693897; doi:10.3390/nu17233684)
Supplement: Supplementary file 1 [file nutrients-17-03684-s001.zip › nutrients-3977904-supplementary.pdf]

SUPPLEMENTARY INFORMATION

Bergamot polyphenols reduce hepatic lipogenesis, while boosting autophagy and short-chain fatty acids production in a murine "cafeteria" model of MASLD.

Concetta Riillo[1], Maddalena Parafati[2], Francesco Crupi[1], Bartosz Fotschki[3], Monica Ragusa[4], Anna Di Vito[5], Chiara Mignogna[5], Vincenzo Mollace[1,6] and Elzbieta Janda* [1,6].

1 Department of Health Sciences, University Magna Graecia, 88100 Catanzaro, Italy.
2 Department of Pharmacodynamics, College of Pharmacy, University of Florida, Gainesville, FL 32610, USA.
3 Institute of Animal Reproduction and Food Research, Polish Academy of Sciences, 10-748 Olsztyn, Poland.
4 Current address: Department of Veterinary Prevention, Provincial Health Authority of Messina, Italy.
5 Department of Experimental and Clinical Medicine, University Magna Graecia, 88100 Catanzaro, Italy.
6 Institute of Research for Food Safety & Health IRC-FSH, University Magna Graecia, 88100 Catanzaro, Italy.

Supplementary Methods

1. BPF dosing

To ensure the daily dosage of 50 mg/kg/mouse, the water consumption over 24 hours was monitored in mL per cage (Vd). To this end, 3 mice from the same cage were weighed to determine the total body mass of mice per cage (Tbm) expressed in kg and subsequently transferred to a metabolic cage for 24 h. To calculate the amount of BPF powder per cage per 2 days (Db) the following formula was used: Db = Vsuf x Dkg /Vd/Tbm, where Dkg is a daily dose per kg of animal mass and in this case 50 mg/kg and Vsuf is a sufficient amount of water (in mL) per 2 days, which should be at least 50% more than 2 x Vd. To prepare the beverage sufficient for 2 days, Db was diluted in Vsuf of drinking water. The beverages were refreshed every two days. To adjust the Db value to the increasing weight of mice, the beverage consumption measurement was repeated as above after 1 and 2 months from the study.

Table S1

Values per 1g/ml of food item	kCal (g/ml)	Fats (g)	Satured fats (g)	Tot. Carb (g)	Sugar (g/g)	Fibre (g/g)
Harlan 2016	3	0.04	0.01	0.64	0.05	0.04
Cola	0.44	0	0	0.11	0,11	0
Vanilla Wafer	5.26	0.27	0.25	0.64	0.34	0.01
Condensed milk	3.22	0.08	0.05	0.55	0.55	0
Sugar	4	0	0	1	1	0
Sweet dried	5.64	0.35	0.18	0.54	0.13	0.01
Sandwich biscuits (cacao)	4.68	0.18	0.1	0.69	0.29	0.03
Bar Choco Caramel	4.76	0.23	0.15	0.63	0.56	0.01
Butter	7.54	0.83	0.58	0.007	0	0
Crackers (brand 1)	4.73	0.2	0.07	0.66	0.12	0.03
Snack Salty (mais)	5.56	0.35	0.15	0.53	0	0.02
Cheese chips (brand 2)	5.32	0.27	0.13	0.65	0.04	0.01
Cheddar cheese	4.03	0.33	0.21	0.12	0.05	0

Table S1. The nutritional facts of foods used to assemble the CAF diet, according to **the Experimental** Design. The brand names of products used can be disclosed on request.

2. Quantitative (q)RT-PCR Analysis

To ensure the daily dosage of 50 mg/kg/mouse, the water consumption over 24 hours was monitored in mL per cage (Vd). To this end, 3 mice from the same cage were weighed to determine the total body mass of mice per cage (Tbm) expressed in kg and subsequently transferred to a metabolic cage for 24 h. To calculate the amount of BPF powder per cage per 2 days (Db) the following formula was used: Db = Vsuf x Dkg /Vd/Tbm, where Dkg is a daily dose per kg of animal mass and in this case 50 mg/kg and Vsuf is a sufficient amount of water (in mL) per 2 days, which should be at least 50% more than 2 x Vd. To prepare the beverage sufficient for 2 days, Db was diluted in Vsuf of drinking water. The beverages were refreshed every two days. To adjust the Db value to the increasing weight of mice, the beverage consumption measurement was repeated as above after 1 and 2 months from the study. Frozen Liver tissues were powdered in a tissue pulverizer (CellCrusher, USA), cooled by liquid nitrogen. Total RNA was extracted from approximately 25 mg of liver tissue using TriFast™ Reagent (Cat No. 30-2010, Peqlab™ a VWR company) according to the manufacturer's instructions. The quantity of RNA was determined using a Multiskan Sky-high combined with a μDrop Plate (ThermoFisher). Subsequently, 5 μg of RNA was treated with DNase I (Cat No. EN0521, ThermoFisher). To evaluate RNA integrity number equivalent (RINe), the RNA Screen Tape (Cat No. 5067-5576, 5067-5578, 5067-5577, Agilent Technologies, Inc.) was used. Total RNA (0.5 μg) was converted to double-stranded cDNA using SuperScript™ VILO™ (Cat No 11754050, Invitrogen™). PCR was performed with primers designed (www.ensembl.org) and listed in Supplementary Material Table S2; hypoxanthine phosphoribosyl transferase 1 (*Hprt-1*) was used as a housekeeping control. Each PCR reaction (total volume of 10 μL) contained 5 μL of Power SYBR™ Green PCR Master Mix (Applied Biosystems, Woolston, UK), primers at a final concentration of 0.2 μmol/L, and 2 μL of cDNA template. Reactions were run in triplicate for each cDNA sample and mouse using a QuantStudio 12K Flex Real-Time PCR System (Applied Biosystems, Woolston, UK). The thermal cycling protocol consisted of an initial denaturation step at 95 °C for 5 min, followed by 40 amplification cycles of 60 °C for 20 s and 72 °C for 45 s. Relative gene expression levels were determined using the 2ΔCT method, with the SC group serving as the control. All tissue samples were analysed in triplicate, and gene expression results were reported as fold changes.

Table S2. List of mouse-specific primers for qRT-PCR analysis of gene expression.

Gene (mouse)		Sequence (5'→3')
Tlr4<br>*Toll-like receptor 4*	*Forward*<br>*Reverse*	TTCTTCTCCTGCCTGACACC<br>TCAAGGGGTTGAAGCTCAGA
Ppara<br>*Peroxisome proliferator-activated receptor alpha*	*Forward*<br>*Reverse*	CCACGAAGCCTACCTGAAGA<br>TTCTCGGCCATACACAAGGT
Srebf1<br>*Sterol regulatory element-binding protein 1*	*Forward*<br>*Reverse*	GCAGTGGTGGTAGTGACTCT<br>TCAGACACAGAAAGGCCAGT
Acly<br>*Adenosine triphosphate citrate lyase*	*Forward*<br>*Reverse*	GAAGCTCATCAAGAAGGCCG<br>GTTGTCCAGCATTCCACCAG
Acaca<br>*Acetyl-CoA carboxylase alpha*	*Forward*<br>*Reverse*	GTTTGGTCGTGACTGCTCTG<br>TTGGCAAGTTTCACTGCACA
Col1a2<br>*Collagen type I alpha 2 chain*	*Forward*<br>*Reverse*	GGAACAAATGGGCTCACTGG<br>CAAGTCCTCTGGCACCTGTA
Hprt<br>*Hypoxanthine phosphoribosyltransferase*	*Forward*<br>*Reverse*	GGATACAGGCCAGACTTTGT<br>GCGCTCATCTTAGGCTTTGT

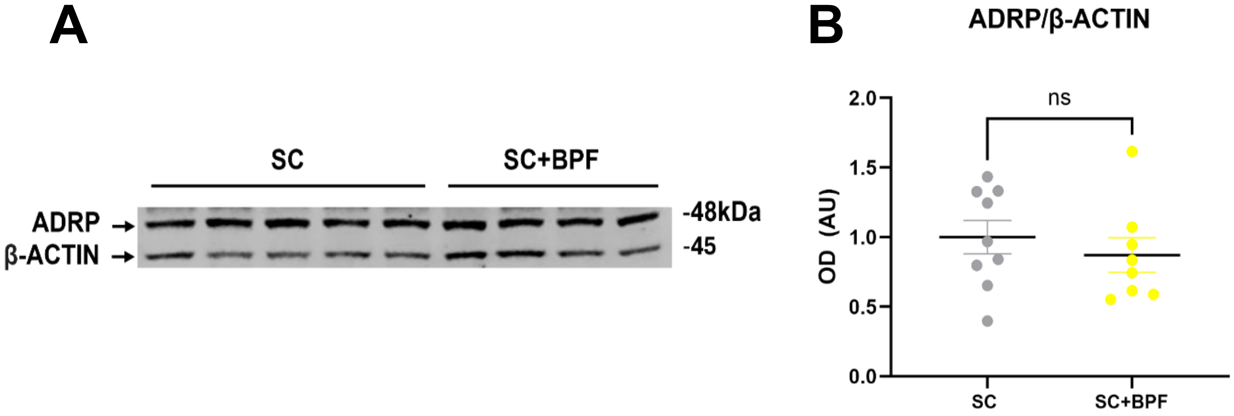

Figure S1. BPF has no statistically significant effect on ADRP expression in C3H mice fed the standard diet SC. (A) Representative blot for ADRP/Perilipin 2 and beta-actin as a loading control showing liver lysates from 4/5 different mice for each group. (B) OD ratio of ADRP to beta-actin expression levels. Data are expressed as the mean ± SEM. Each lysate was loaded twice on a NU-PAGE 4-12% Bis -Tris gel and the data reanalyzed. Statistical analysis: One-way ANOVA followed by Tukey's multiple comparisons test; ns = not significant.
